# Effect of Rotavirus Infection and 2′-Fucosyllactose Administration on Rat Intestinal Gene Expression

**DOI:** 10.3390/nu15081996

**Published:** 2023-04-21

**Authors:** Laura Sáez-Fuertes, Ignasi Azagra-Boronat, Malén Massot-Cladera, Karen Knipping, Johan Garssen, Àngels Franch, Margarida Castell, Francisco J. Pérez-Cano, María J. Rodríguez-Lagunas

**Affiliations:** 1 Physiology Section, Department of Biochemistry and Physiology, Faculty of Pharmacy and Food Science, University of Barcelona (UB), 08028 Barcelona, Spain; 2 Nutrition and Food Safety Research Institute (INSA-UB), 08921 Santa Coloma de Gramenet, Spain; 3 Danone Nutricia Research, 3584 CT Utrecht, The Netherlands; 4 Division of Pharmacology, Utrecht Institute for Pharmaceutical Sciences, Faculty of Science, Utrecht University, 3584 CA Utrecht, The Netherlands

**Keywords:** rotavirus, infection, 2-fucosyllactose, oligosaccharide

## Abstract

Viral infections are described as modifying host gene expression; however, there is limited insight regarding rotavirus (RV) infections. This study aimed to assess the changes in intestinal gene expression after RV infection in a preclinical model, and the effect of 2-fucosyllactose (2′-FL) on this process. From days 2 to 8 of life, rats were supplemented with the dietary oligosaccharide 2′-FL or vehicle. In addition, an RV was inoculated on day 5 to nonsupplemented animals (RV group) and to 2′-FL-fed animals (RV+2′-FL group). Incidence and severity of diarrhea were established. A portion from the middle part of the small intestine was excised for gene expression analysis by microarray kit and qPCR. In nonsupplemented animals, RV-induced diarrhea upregulated host antiviral genes (e.g., *Oas1a*, *Irf7*, *Ifi44*, *Isg15*) and downregulated several genes involved in absorptive processes and intestinal maturation (e.g., *Onecut2*, and *Ccl19*). The 2′-FL-supplemented and infected animals had less diarrhea; however, their gene expression was affected in a similar way as the control-infected animals, with the exception of some immunity/maturation markers that were differentially expressed (e.g., *Ccl12* and *Afp*). Overall, assessing the expression of these key genes may be useful in the evaluation of the efficacy of nutritional interventions or treatments for RV infection.

## 1. Introduction

The World Health Organization (WHO) has established that human milk (HM) is the most suitable option to meet nutritional requirements during the first period of life [1]. HM starts to be produced during the late gestational phase, and its composition changes throughout the lactation period [2]. Colostrum is the first milk produced after delivery and is rich in immunological components [2]. Five days postpartum, milk composition starts to change to adapt to the nutritional and developmental needs of the infant, which is called transitional milk. Then, after two to three weeks postpartum, the human milk composition is considered mature [3].

HM has several components, which, in addition to providing nutrition, contribute to protecting infants from diseases. These components are classified into macronutrients (~99%) and micronutrients (~1%). Macronutrients include water, carbohydrates, proteins, and lipids [4]. During the milk maturation process, the proportions of macronutrients such as proteins, fats, and carbohydrates vary [2]. Within carbohydrates, human milk oligosaccharides (HMOs) are a complex group of indigestible carbohydrates, with more than 200 structures identified so far; they present prebiotic activities [5] due to their beneficial effects on the host microbiota [6]. *Bifidobacteria* and *Bacteroidetes* are the main microorganisms able to metabolize HMOs [7]. The most abundant HMO in human milk is 2′-fucosyllactose (2′-FL). This HMO has been widely studied to evaluate its impact on offspring development and its potential to reduce infections during early life [8,9,10]. In this regard, this HMO has been included in infant formula to come closer to HM composition and improve and modulate the immune system [11] and gut microbiota [12].

Rotavirus (RVs) is a nonenveloped double-stranded RNA (dsRNA) virus whose genome is protected by a 3-capsid structure and has the information needed for six structural proteins and five nonstructural proteins. Viral particles are able to reach the small intestine and replicate within the enterocytes [13], which disrupts the absorptive functions and fluid secretion, inducing severe dehydration and diarrhea in children, mainly before 5 years of age [14]. Once the enterocytes are infected, different cellular mechanisms recognize viral components in endosomal compartments (Toll-like receptor (*TLR*) 3) and in the cytoplasm (retinoic acid-inducible gene I (*RIG-I*) and melanoma differentiation-associated gene-5 (*MAD-5*)) [15,16]. To sum up, after RV particles reach the small intestine and infect enterocytes, the immune system cells will trigger an immunological response by changing gene expression profiles to produce cytokines and chemokines for the recruitment of immune cells needed to attack and remove the viruses [15].

Due to the high incidence of RV infections in infants, multiple studies have been conducted searching for alternatives to reduce RV infections and/or improve immune resistance to the virus. Among the options, dietary supplementations with probiotics and prebiotics have proven to impact and reduce RV-induced diarrhea [17,18]. To explain and understand the reduction in RV-induced diarrhea, molecular mechanisms have been studied, including changes in intestinal gene expression of particular receptors involved in viral resistance and immunity, such as the TLR, mucins, or tight junction proteins, among others [10]. However, concrete data on gene expression in an RV infection model and its modulation by dietary components have not been gathered.

In a previous study, it was demonstrated that daily supplementation of 2′-FL prevented several pathological effects in an RV infection rat model [10], and some mechanisms were suggested. The aim of this study was to evaluate the impact on small intestine gene expression after supplementation with 2′-FL during early life in an RV infection model. This study aims to explain the molecular pathways activated during RV infection and the mechanism underlying the reduction in RV-induced diarrhea after 2′-FL supplementation in rats.

## 2. Materials and Methods

### 2.1. Animals

Twelve G15 pregnant Lewis rats were obtained from Janvier Labs (Le Genest-Saint-Isle, France). The day of birth was considered as day 1 of life, and pups were randomly distributed into the four experimental groups. Litters were unified to eight pups with free access to maternal milk, and dams were fed ad libitum. From day 2 to day 16 of life, pups were separated daily from dams for oral administration and then reunited again.

From the day of birth, all litters were housed in cages in an isolated room under biosecurity level 2 conditions at the Animal Facility of the Faculty of Pharmacy and Food Science at the University of Barcelona (UB). Animals were under temperature and humidity-controlled conditions in a 12 h/12 h light/dark cycle [19,20,21]. All procedures of care and use of animals were approved and conducted in accordance with the Ethical Committee of the UB and Catalonia Government (CEEA-UB, Ref. 74/05 and DAAM 3046, respectively).

### 2.2. Experimental Design and Sample Collection

Newborn rats were distributed into four study groups (each group constituted by three litters of 8 pups each): reference (REF) group, 2′-fucosyllactose (2′-FL) group, rotavirus-infected (RV) group and rotavirus-infected group with 2′-FL supplementation (RV + 2′-FL). The number of animals per group was calculated taking into account that there was at least one animal from the three different litters in each group, due to the strong maternal influence in the process [22].

Suckling rats were orally administered in the first hours of the light phase, as previously described by Rigo-Adrover et al. [23]. The 2′-FL group and the RV + 2′-FL were supplemented from the day of birth with 0.2 g of 2′-FL/100 g of body weight (4.5 μL/g/day of a 4.5 g/mL solution), while REF and RV groups were administered with a matched volume of water [10].

The RV (simian SA-11) strain inoculated to animals was provided by the Virus Entèrics group from the UB. The infection was performed on day 5 of life at a dose of 4 × 10^8^ tissue culture infectious dose 50 [TCID_50_]/rat, similar to previous studies [24]. To assess the impact of RV infection, fecal samples and clinical evaluation were performed [17]. Fecal samples were obtained daily by massaging the abdomen and scored to analyze the severity of diarrhea. The scoring scale (diarrhea index, DI) ranged from 1 to 4, with 1 being normal feces and 4 being watery feces.

At day 8 of life, during the maximum peak of diarrhea, selected pups from each experimental group and at least one from each litter (*n* = 4) were anesthetized by using a mix of ketamine (90 mg/kg) and xylazine (10 mg/kg). After the euthanasia, exsanguination by cardiac puncture was performed, and a 1 cm section of the central part of the small intestine was collected in RNA later to analyze gene expression.

### 2.3. RNA Extraction and Microarray Procedure

Representative samples were homogenized as Massot-Cladera et al. described [20]. Afterward, RNA isolation was performed following the manufacturer’s recommendations by using RNAeasy Mini Kit (Qiagen, Madrid, Spain) and quantified with a NanoDrop spectrophotometer and NanoDrop IVD-1000 v.3.1.2 software (NanoDrop Technologies, Wilmington, DE, USA). To analyze the results, Agilent 2100 Bioanalyzer with the RNA 6000 LabChip kit was used. Only samples with RNA integrity number ≥ 9 were selected.

The study of the differential expression profiling was carried out with a SurePrint-G3 Rat GE 8 × 60 K microarray kit (ID 028279, Agilent Technologies, Madrid, Spain), following a loop experimental design in which a pairwise comparison was performed in collaboration with BA Microarray (Alicante, Spain). Quadruplicate samples at day 8 of the design for each experimental condition (REF, RV, and RV+2′-FL) were used, and dye swaps (Cy3 and Cy5) were performed on the RNA amplified from each sample. RNA quality was assessed using a TapeStation (Agilent Technologies). RNA concentration and dye incorporation were measured using a UV-VIS spectrophotometer (Nanodrop 1000, Agilent Technologies, Wilmington, DE, USA). Labeling and hybridization to microarray were conducted following the manufacturer’s two-color protocol (Two-Color Microarray-Based Gene Expression Analysis v. 6.5, Agilent Technologies), using LowInput QuickAmp Labeling Kit and Agilent Microarray Hybridization Chamber Kit for labeling and hybridization, respectively. Microarray chips were then washed and immediately scanned using a DNA microarray scanner (Model G2505C, Agilent Technologies) by the Genetic Diagnostic Bioarray facilities (Bioarray, Alicante, Spain).

### 2.4. Microarray Data Analysis

Data extraction was performed with Agilent Feature Extraction Software v.10.7 (Agilent Technologies). Bioinformatic analysis was performed with Bioconductor software under R environment, using the following packages: limma (v.3.16.1) for background correction and normalization; Marray and pcaMethods for quality control plots; RankProd for differential expression; and finally GOstats (v.2.26.0.) and GSEABase for gene ontology functional analysis. The latest gene annotations available were used. Raw feature intensities were background-corrected using the normexp background correction algorithm. Within-array normalization was performed using spatial- and intensity-dependent loess. Quantile normalization was used to normalize between arrays. The expression of each gene was reported as the base 2 logarithm of ratio of the value obtained of each condition relative to the control condition (REF group or RV group). A gene was considered differentially expressed if it displayed a PFP (percentage of false prediction, equivalent to the false discovery rate, FDR) of less than 0.05 by rank product nonparametric method (RankProd). Finally, up- and downregulated genes were analyzed in terms of gene ontology by using a hypergeometric analysis (GOStats).

### 2.5. Validation of Gene Expression by Real-Time PCR

Two µg of total RNA were converted to cDNA. Specific PCR TaqMan^®^ primers and probes (Applied Biosystems, AB, Weiterstadt, Germany) were used to measure selected targets, depending on the results raised. In addition to the three studied groups (RV, RV, and RV+2′-FL), the PCR was also performed in the 2′-FL group in order to observe whether some effects induced by 2′-FL in the context of infection (RV+2′-FL) were also observed in a health context (2′-FL). Quantitative real-time PCR assays were performed for eight samples/group using an ABI PRISM 7900HT Sequence Detection System (AB). The specific PCR TaqMan^®^ primers (AB) were *Isg15* (Rn01519614_m1_I), *Oas1a* (Rn04219673_m1_I), *Irf7* (Rn01450778_g1_I), *Ifi44* (Rn01523064_m1_I), *Ccl19* (Rn01439563_m1_I), *Ccl12* (Rn01464638_m1_I), *OneCut2* (Rn01265320_m1_I), and *Afp* (Rn00560661_m1_I). Quantification of the genes studied was normalized to the housekeeping gene *Gusb* (Rn00566655_m1, I). The SDS v2.4 software (AB) was used to analyze the expression data. Results were expressed as the fold change in the amount of target mRNA relative to the endogenous control expression calculated using the standard 2-∆∆Ct method for the different experimental groups in relation to values from the REF or RV group, which represent a onefold change in gene expression.

### 2.6. Statistical Analysis

PCR results were statistically analyzed using the software package SPSS 22.0 (SPSS, Inc., Chicago, IL, USA). To evaluate variance equality and normal distribution, Levene’s and Kolmogorov–Smirnov tests were conducted. Kruskal–Wallis and Mann–Whitney U (MWU) tests were performed when results did not follow a normal and equal distribution to assess significant differences among groups (*p* < 0.05). To compare the frequencies of diarrhea incidence (%DA), the chi-square test was performed. Results are expressed as mean ± standard error of the mean (S.E.M).

## 3. Results

### 3.1. Clinical Results

**Figure 1:**
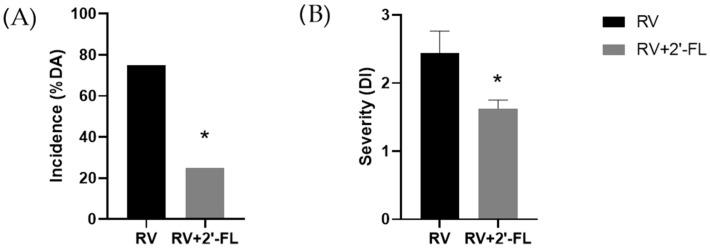
Preventive effect of 2′-FL on the RV infection. (**A**) Incidence (%DA, Diarrheic Animals) and (**B**) Severity (DI, Diarrhoea Index) on day 8 of the selected animals of the array study (*n* = 4, from three different litters). Results are expressed as unique values of % for incidence (derived from all animals each day) and as mean ± S.E.M. for severity. Statistical differences: * *p* < 0.05 compared to RV group by chi-square test and by MWU test, respectively.

Clinical evaluation carried out at the peak of the infection (day 8) by scoring feces from 1 to 4 allowed the calculation of the incidence and the severity of RV-induced diarrhea in the subgroup of animals selected for the gene expression analysis in the present study (Figure 1). The results showed a lower percentage of animals having diarrhea (%DA) and lower severity (*p* < 0.05) in the RV+2′-FL group compared to the RV group, demonstrating that supplementation with 2′-FL ameliorates rotavirus-induced diarrhea in similar and representative proportions of those obtained from the full cohort in previous studies [10].

### 3.2. RV Effect on Overall Intestinal Rat Gene Expression

**Figure 2:**
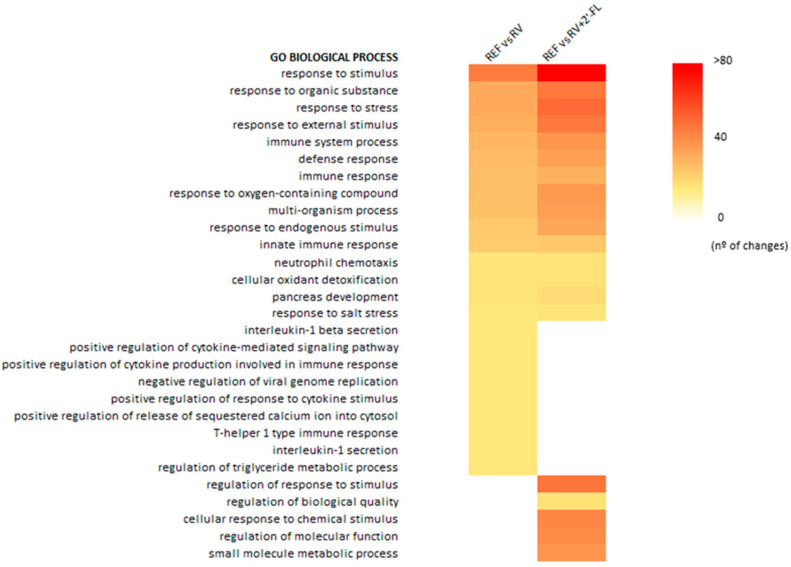
List of the gene ontology (GO) pathways in which higher numbers of statistically affected genes are counted in the array due to the RV infection with respect to the REF group (*n* = 4/group).

The infection with RV in both groups (RV and RV+2′-FL) impacted the intestinal gene expression during the peak of diarrhea by means of the overall gene ontology (GO) approximation (Figure 2). Both groups displayed a number of genes being statistically modified due to the infection. Most of these genes were related to stress and immunity GO biological processes.

Among the upregulated GO processes, the defense response to the virus, the cellular response to the type-I interferon, and the positive regulation of innate immune response were demonstrated. In contrast, the main downregulated GO processes were associated with the positive regulation of neutrophil chemotaxis and intestinal absorption. These results are in line with the main effects of RV infection in the small intestine.

The RV+2′-FL affected not only a higher number of genes in pathways already modified by the RV but also induced changes in other pathways, such as the regulation of response to stimulus, biological quality, molecular function, and small molecule metabolic processes (Figure 2).

### 3.3. Gene Expression Changes Due to RV Infection and 2′-FL Supplementation

In order to evaluate the impact of RV infection on gene expression in the small intestine of 8-day-old pups, the fold change for the genes that were modified significantly was studied (Table 1). The genes are ordered based on the fold change comparing RV vs. REF and RV+2′FL vs. REF, depending on whether they were upregulated (Table 1A) or downregulated (Table 1B)**.** RV infection significantly upregulated the expression of several genes (*n* = 30), which were mainly involved in the defense response against the virus and the innate immune response, such as *Oas1a* (*2’-5’ oligoadenylate synthetase 1A*), *Oas1k* (*2’-5’ oligoadenylate synthetase 1K*), and *Usp18* (*ubiquitin specific peptidase 18*) genes. Another upregulated gene was *Rpl391* (*60S ribosomal protein L39*), involved in innate immune responses in the mucosa and in viral transcription processes. In contrast, many genes were downregulated (*n* = 40) by the RV infection, such as *Tmprss15* (*transmembrane serine protease 15*), *Onecut2* (*one cut homeobox 2*), and *Afp* (*alpha-fetoprotein*). These genes are involved in nutrient digestion processes and produced in the duodenum (*Tmprss15*) or associated with anatomical morphogenesis.

In addition, the effect of the supplementation with 2′-FL was evaluated in RV infection. The main up- and downregulated genes are also shown in Table 1A,B, respectively. In these two lists, it can be observed that many changes were shared in both groups, and similarly to what was found without the supplementation, *Oas1a* and *Oas1k* genes were the most abundant upregulated genes. Comparing the most downregulated genes, it was observed that *Tmprss15* and *Onecut2* followed the same expression pattern as in the RV group, whereas *Afp* was highly downregulated after the supplementation with 2′-FL in RV infection. Only three genes were differentially expressed between groups: *Rpl391* (upregulated in the RV group but not affected in the RV+2′-FL group), *Ccl19* (*C-C motif chemokine ligand 19*, downregulated in the RV group but not affected in the RV+2′-FL group), and *Afp*, the downregulation of which is higher in the case of the RV+2′-FL group.

**Figure 3:**
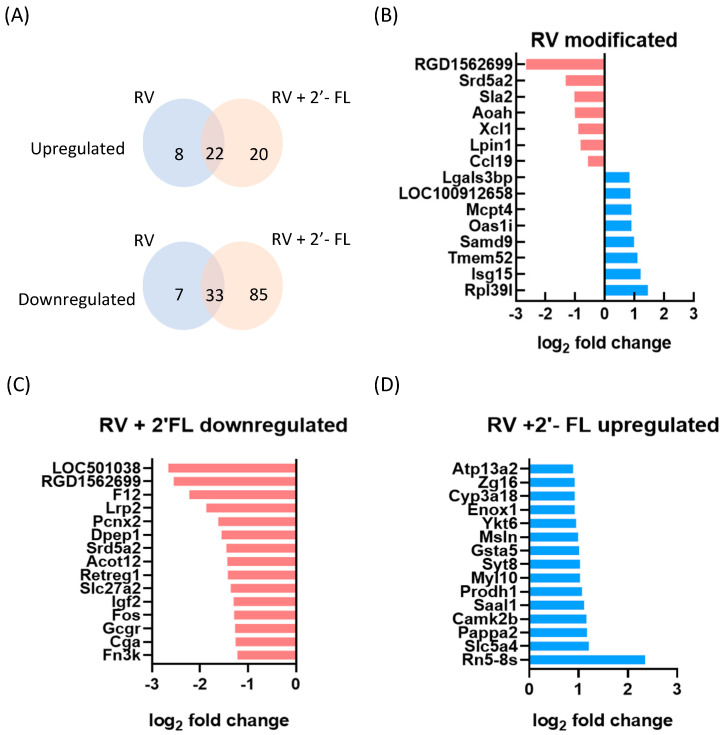
Impact of RV infection and 2′-FL on intestinal gene expression. (**A**) Venn diagram displaying statistically differential up- and downregulated genes with respect to the REF group; (**B**) genes exclusively modified in the RV group; (**C**) the 15 (from the 20) most downregulated genes exclusively modified in the RV+2′-FL group; and (**D**) the 15 (from the 85) most upregulated genes exclusively modified in the RV+2′-FL group (*n* = 4/group).

Additionally, Figure 3 shows different approaches to better understand the impact of RV infection and 2′-FL on intestinal gene expression. First, the Venn diagram (Figure 3A) shows that RV infection modifies a total of 70 genes (30 up- and 40 downregulated), with 55 genes (22 upregulated and 33 downregulated) being shared in the RV and RV+2′-FL groups.

In order to evaluate which genes were exclusively modified by RV infection, a plot displaying these genes was created (Figure 3B). It can be observed that *Ccl19* is found among the most downregulated genes, whereas *Isg15* (*Isg15 ubiquitin-like modifier*) and *Rpl39* are the most upregulated genes, as already observed in Table 1.

The Venn diagram also shows that 105 genes were exclusively modified in the RV+2′-FL group (20 genes up- and 85 downregulated). To zoom in on the exclusive changes due to the 2′-FL intervention, the 15 most affected genes in each case were also identified (Figure 3C,D). The three most upregulated genes exclusively present in the RV+2′-FL group were the uncharacterized proteins LOC501038 and RGD1562699 and F12, also known as coagulation factor XII. On the other hand, the three most downregulated genes exclusively present in the RV+2-’FL group were the ribosomal RNA gene *Rn5-8s*, the *solute carrier family 5 member 4* (SLC5A4), also known as SGLT3, and the enzyme that cleaves an insulin-like growth-factor-binding protein, also known as Pappa2 (pregnancy-associated plasma protein-A2).

**Figure 4:**
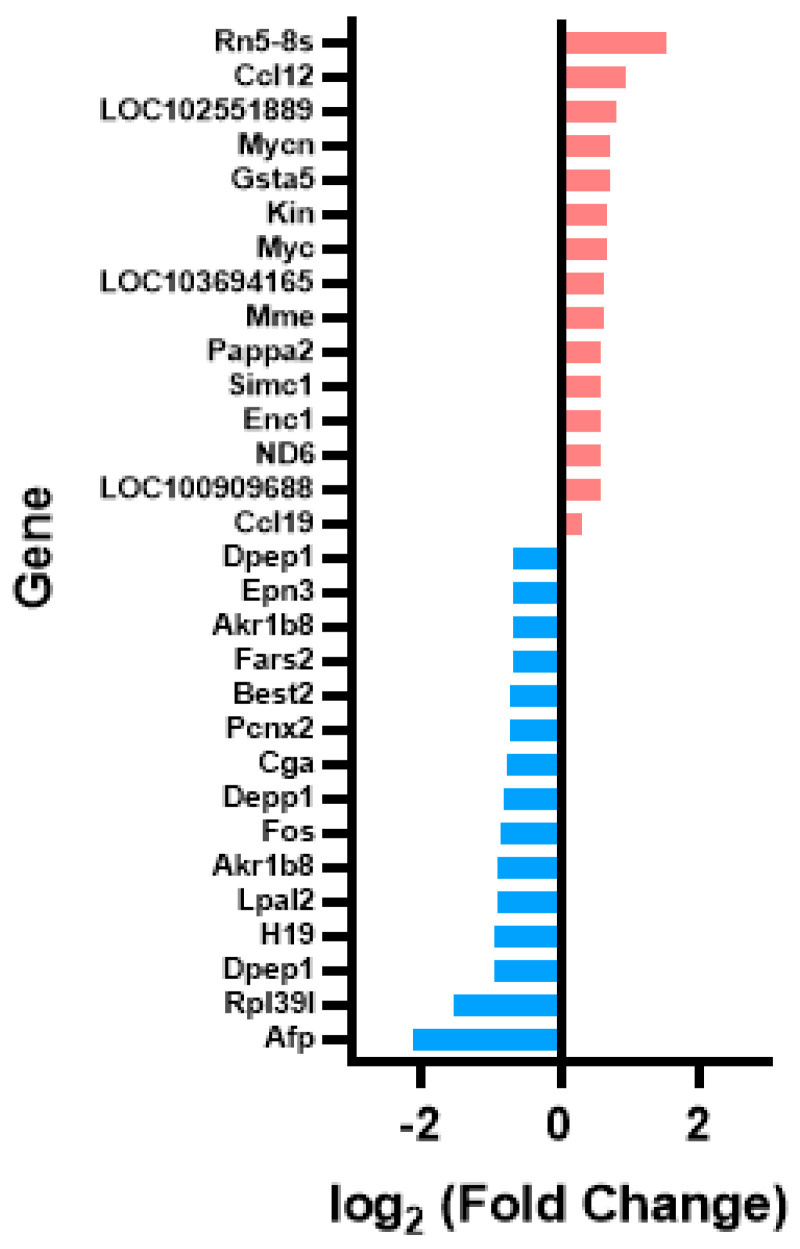
Changes in gene expression between RV and RV+2′-FL groups. The 15 most upregulated genes (red) and the 15 most downregulated genes (blue) comparing the RV+2′-FL group vs. RV group (*n* = 4/group).

Finally, as the previous results were found by comparing both infected groups with respect to the REF group, another approach was taken to evaluate the impact of the 2′-FL under RV conditions (Figure 4). For this, the 15 most upregulated and downregulated genes of the RV+2′-FL group were evaluated compared to the RV group. The results confirmed previous data showing that, whereas *Ccl12* (chemokine (C-C motif) ligand 12) and Rn5-8s were the most upregulated genes, *Afp* and *Rpl39l* were the most downregulated genes with the 2′-FL supplementation.

### 3.4. PCR Confirmation of Key Genes

**Figure 5:**
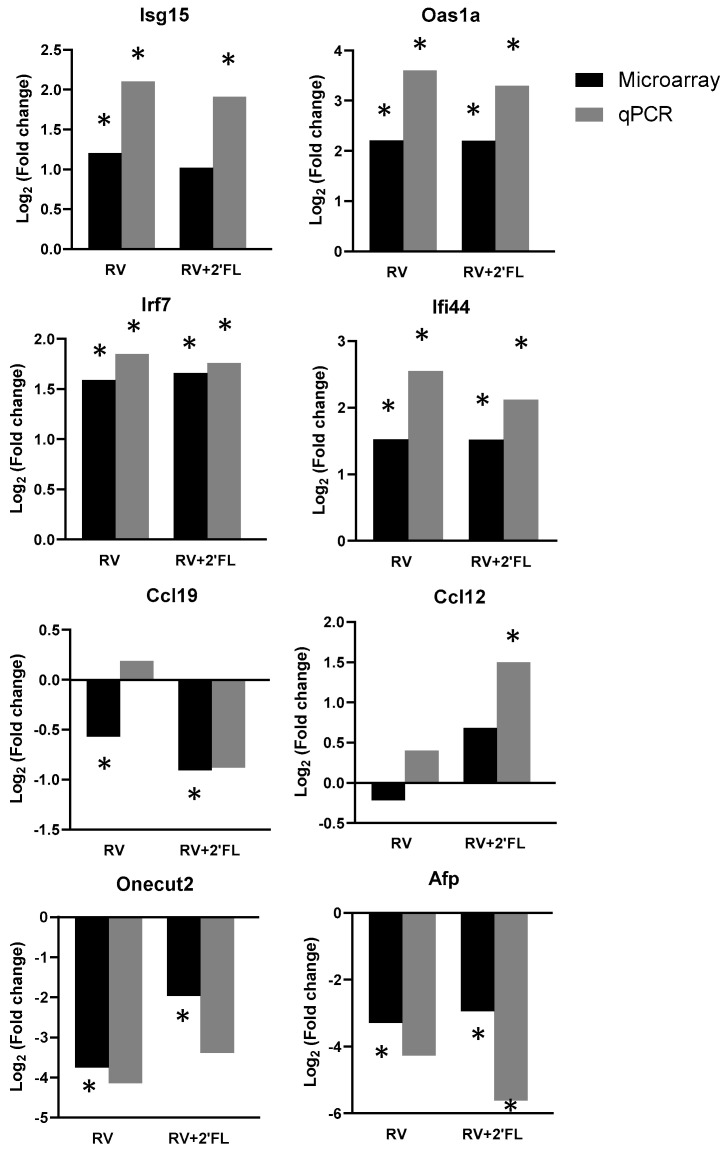
Fold change in selected target genes with respect to REF group by the array results and by Taqman PCR (*n* = 4/group in the array and *n* = 8/group in the qPCR). Statistical differences: * *p* < 0.05 vs. REF.

To confirm the array results, qPCR of key genes was performed (Figure 5). The main upregulated genes in both groups were *Isg15*, *Oas1a*, *Irf7*, and *Ifi44*. All of them are involved in the type-I interferon pathway and were confirmed by qPCR. The gene expression of two chemokines was also analyzed (*Ccl19* and *Ccl12*). Only *Ccl12* was confirmed in the RV+2′-FL group by qPCR. *OneCut2* and *Afp* genes were highly modified in microarray results in both groups. However, qPCR results only confirmed *Afp* gene expression in the RV+2′-FL group.

### 3.5. Gene Expression Changes with 2′-FL Supplementation

**Figure 6:**
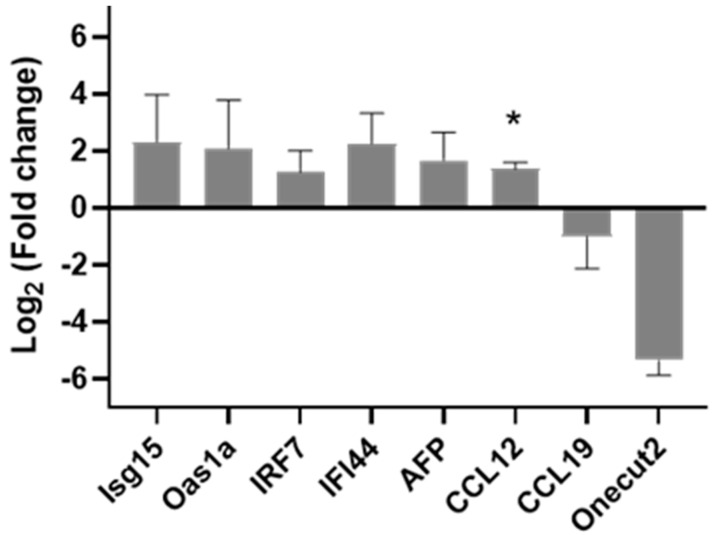
Fold change in selected target genes in the 2′-FL group with respect to REF by Taqman PCR (*n* = 4–8/group). Statistical differences: * *p* < 0.05 vs. REF.

After the microarray and qPCR evaluation of the RV and RV+2′-FL group, the most interesting genes were analyzed in the 2′-FL group by qPCR as well (Figure 6).

The results from Figure 6 showed that the expression of *Ccl12* was increased with 2′-FL supplementation, similar to what was found in the RV+2-FL. Apart from Ccl12, the upregulation of the gene expression of *Isg15*, *Oas1a*, *Irf7*, and *Ifi44* and the downregulation of *Ccl19* and *OneCut2* after the 2′-FL supplementation were similar to the RV+2′-FL group.

## 4. Discussion

Since 2020, COVID-19 has placed viral infections and the necessity to understand its physiopathology in the spotlight. However, it should not be forgotten that many other viruses also affect human health. Among them, RVs are one of the most infectious agents in children, especially in low-income countries. In this context, more studies to identify underlying mechanisms for RV-induced pathophysiology are essential and aim to produce better tools to prevent and treat RV infections in humans.

Previous in vitro and in vivo studies have reported that RV particles can change the expression of several genes, especially in the small intestine [10,15]. The majority of these studies are focused on potential and specific target genes associated with inflammation [25]. In this regard, it might be that specific dietary interventions using, e.g., pro- or prebiotics or even specific HMOs such as 2′-FL can interfere with these processes, leading to changes in severity and incidence of RV-related health features (i.e., diarrhea, inflammation, and tissue damage) [26,27,28].

To elaborate on our previous study showing the positive effect of 2′-FL on RV-induced diarrhea, in the present study, we moved into more molecular details using specific microarray technologies. In the end, we aimed to evaluate the role of RV infection in gene modifications and the potential of 2′-FL supplementation to reduce the impact of RV infection. 2′-FL is the most abundant oligosaccharide in human milk and has been associated with beneficial functions, including the reduction in inflammatory responses by modulating gene expression [9,10] and intestinal microbiota [29]. Our findings indicate that supplementation with 2′-FL to rats during early life attenuates the incidence and severity of the RV infection. These results are in line with previous nutritional interventions in our laboratory study [10] and by others where a mix of HMOs was used [30,31].

Having demonstrated that specific dietary supplementation reduces viral infections, different approaches were utilized to evaluate its impact on the genetic expression located in the small intestine and in order to create a mechanistic understanding. As can be observed in the Venn diagrams, RV infection induced several changes in intestinal gene expression. However, the supplementation with 2′-FL under RV infection induced even more changes in the small intestine tract at this gene level. These exclusively modified genes after nutritional supplementation under RV conditions may be associated with the protective role of 2′-FL in these preclinical disease models.

In general, RV infection modifies different genes grouped in GO biological processes. The upregulated GO processes are involved in the activation of viral immunity mediated by type-I interferon. In contrast, the most downregulated GO processes are linked to intestinal absorption but also neutrophil chemotaxis. These results are in accordance with previous reports of intestinal infections [32,33].

The genes that are the most modified by this RV infection are linked to GO biological processes of the response to external stimulus, and are especially associated with viruses aimed to activate viral immunity. In this type of infection, the RV viral particles are recognized by pattern recognition receptors (PRRs) and trigger the activation of the innate immune viral response, inducing the expression of pro-inflammatory genes. One of the main pro-inflammatory-activated genes is interferon, including type-I and type-III [34]. During viral infections, both interferon families, interferon-stimulated genes (ISGs) and interferon-regulatory factors (IRFs), collaborate to produce an efficient virus clearance [35]. Type-I (α/β) IFN and type-III (λ) *IFN* coordinate and overlap some of their functions during viral infections, but there are specific functions associated with each one. To date, *IFN*-λ is expressed in intestinal epithelial cells and displays a protective role in mucosal surfaces, while *IFN*-α/β is expressed in systemic tissues and organs (liver, spleen, and kidney), avoiding the systemic spread of the infection [34]. However, changes in *IFN* gene expression at the intestinal level were not found in this model. However, the genes involved in IFN pathways, such as the OAS family, *Irf7*, *Ifi44,* and *Isg15,* were found to be affected by RV infection. The OAS (*Oas1*, *Oas2*, *Oas3,* and *OasL*) family are IFN-stimulated proteins that are highly induced by type-I *IFN*. This family is involved in viral RNA degradation to facilitate viral recognition by *RIG-I* and *MDA5* pathways. In particular, *Oas1a* has been associated with the induction of IFN signals [36]. Our microarray results showed that the most upregulated gene was *Oas1a*, indicating that its induction is involved in the inflammatory state and the IFN activation pathway initiated. The RV+2′-FL showed similar results to the RV group, suggesting that the reduction in RV-induced diarrhea in the RV+2′-FL group is not due to changes in this particular gene.

Similarly to the OAS family, Ifi44 is one of the first ISGs after viral infections to stimulate PPRs. Its expression results in the activation of different transcription factors, such as *Irf3* and *Irf7*, which promotes IFN signaling [37]. In this context, it is suggested that *Ifi44* acts in the first defense line of the viral immune response, while *Irf7* is involved in the later stage of IFN induction [38]. *Irf7* stimulation will contribute to the activation of innate immune responses. In our case, microarray and qPCR results showed that RV and RV+2′FL induced the gene expression of *Ifi44* and *Irf7* in the small intestine, thus activating antiviral responses. Comparing the RV and RV+2′-FL groups, no statistical differences were observed between them in terms of *Ifi44* or *Irf7* gene expression under RV conditions. Although our results are in line with previous studies [37], it is important to mention that many viruses, including RV, have developed multiple evasion mechanisms. The main evasion pathway relies on the degradation of different IRFs, such as *Irf3*, *Irf5*, and *Irf7* [39], which have not been found to be statistically modified in this approach either.

Another upregulated gene by RV infection was *Isg15*, which works together with *Usp18*. The mechanism of action of the *Isg15* gene is based on the conjugation of Isg15 with different molecules (ISGylation), which stimulates the IFN type-I pathway [40]. In the beginning, Usp18 was associated with a reverse function of ISGylation, reducing the inflammatory IFN pathways [41]. However, a later study reported that Usp18 plays a double role in inducing or blocking IFN signaling. *Usp18* can directly activate the JAK/STAT signaling pathway to promote IFN responses [42] or may bind to interferon receptor 2 (IFNAR2) and inhibit the JAK/STAT [43]. Considering the above, the overexpression of *Isg15* promotes IFN signals. In contrast, the role of *Usp18* is not yet clear. Our results indicate that RV infection upregulates the expression of *Isg15,* whereas the supplementation of 2′-FL is not able to modify its expression with or without RV conditions. With regard to *Usp18* mRNA expression, both RV and RV+2′-FL increased it. These results are in accordance with Ye et al. [42] under dengue infection. Nevertheless, further studies are needed to elucidate the positive or negative impact on RV clearance.

In addition to the changes in genes involved in the IFN pathway, many other genes were modified, including chemokines, transmembrane proteins, and ribosomal proteins.

After viral or bacterial infections, different chemokines collaborate to induce an effective response. To fight against pathogens, the expression of pro-inflammatory, anti-inflammatory, or homeostatic chemokines can be stimulated. Among the changes in the homeostatic chemokines, *Ccl12* and *Ccl19* are included [44]. They play a critical role in the first line of defense, and their functions are linked with immune cell recruitment [45]. Our microarray and qPCR results showed that *Ccl19* was reduced under RV conditions, suggesting that the downregulation of this gene limits the mechanisms of action of the immune system to recruit immune cells. In contrast, the microarray results revealed that *Ccl12* was upregulated in the RV+2′-FL, and the qPCR results of the 2′-FL group showed that the expression of this gene was also upregulated in noninfected conditions. Combining these results, it can be confirmed that the supplementation with 2′-FL during early life is able to modify the expression of *Ccl12* in the small intestine to modulate immunity and enhance the defense against pathogens such as RV.

*Tmprss15* was the most downregulated gene in both the RV and RV+2′FL groups. This gene is a transmembrane protease involved in trypsin metabolism. *Tmprss15* collaborates in the proliferation process during viral infections, such as the influenza A virus [46]. Additionally, a deficiency of this enzyme is associated with enterokinase deficiency (EDK), characterized by severe chronic diarrhea after birth [47]. Similarly to EDK, the main manifestation of RV infection is diarrhea, suggesting that diarrhea is highly linked to the downregulation of *Tmprss15*. In our case, the supplementation of 2′-FL reduced RV-induced diarrhea, but the expression of *Tmprss15* did not change, indicating the modulation of these genes cannot explain the diarrhea reduction by the 2′-FL supplementation.

Comparable to *Tmprss15*, the expression of *OneCut2* was highly downregulated in the RV and RV+2′-FL groups. The *OneCut* family of transcription factors is involved in organ development, such as that of the gut endoderm, liver, and pancreas [48]. Since the identification of this family, most of the related studies have focused on neuronal and liver development diseases [49]. No information is known about the relationship between *OneCut2* and viral infections in early life. For this reason, further studies are needed to clarify its relation and whether infection can affect intestinal development.

Another upregulated gene was *Pappa2*. The down-expression of this gene has been associated with changes in growth parameters [50,51]. In our case, the supplementation with 2′-FL under RV conditions upregulated the expression of this gene. However, the previously published results showed that the growth evolution of these animals was not affected during the nutritional intervention [10,52], and thus did not affect postnatal growth.

Finally, among the gene expression modifications, the genes that were differentially modified comparing RV and RV+2′-FL groups will be discussed. The expression of *Rpl39l* and Afp genes was differentially influenced by 2′-FL under RV conditions. To date, there have been no correlations between *Rpl39l* and *Afp* expression patterns and viral infections. Changes in the expression of *Rp39l* have been associated with hepatocellular carcinoma tumors [53], while *Afp* expression is important during fetal growth, contributing to appropriate gastrointestinal development [54]. Our microarray results demonstrated that 2′-FL attenuates the overexpression of *Rpl39* and boosts the down-expression of *Afp*. Although little information is known about the relation between *Rpl39l*, *Afp,* and RV infections, we suggest that the mechanism of action of 2′-FL under RV conditions to reduce diarrhea is linked to intestinal development. However, more studies are required to clarify the role of 2′-FL in early life, including its mechanism of action to mitigate the impact of RV infections.

Thus, further research is needed to elucidate the role of 2′-FL on intestinal maturation and its influence on *Afp*, *OneCut,* and *Pappa2* gene expression. Additionally, the impact of 2′-FL supplementation on the expression of genes involved in the immune response (chemokines and transmembrane proteins) will provide a better understanding of its protective effects. All these analyses will contribute to completing the gaps in the knowledge of 2′-FL.

A current limitation of this research is the lack of a wide gene expression study of the 2′-FL in early life under physiological development. Only the most interesting genes raised by the microarray were studied. 

## 5. Conclusions

In this work, we identified the main gene expression modifications during RV infections in a preclinical disease model. The effect of the supplementation with 2′-FL during early life seems to be linked to its ability to attenuate RV-induced diarrhea through changes in different identified genes involved in the IFN pathway and intestine maturation involved in the RV pathology. However, as this study is a preclinical approach, more data and investigations, including human studies, are needed to validate these effects and those from other HMOs in viral infections.

## Figures and Tables

**Table 1:** List of partial genes (A) upregulated and (B) downregulated.

(A) Upregulated			(B) Downregulated		
Gene	RV	RV+2′-FL	Gene	RV	RV+2′-FL
*Oas1a*	2.21	2.04	*Ccl19*	−0.57 *	-
*Oas1k*	2.1	1.87	*Slpi*	−0.77	−1.06
*Usp18*	2.06	1.75	*Lpin1*	−0.82	-
*Zbp1*	1.71	1.79	*Alpk3*	−0.83	−1.02
*Irf7*	1.59	1.51	*LOC103691469*	−0.84	−1.08
*Tmigd1*	1.53	1.89	*Kng2l1*	−0.85	−1.01
*Ifi44*	1.52	1.38	*Gkap1*	−0.87	−0.96
*Cfb*	1.51	1.58	*S100g*	−0.88	−0.97
*Rpl39l*	1.45 *	-	*Xcl1*	−0.9	-
*Dhx58*	1.4	1.2	*Abca8a*	−0.94	−1.14
*Ifi27*	1.32	1.44	*Gpcpd1*	−0.95	−0.93
*LOC679368*	1.24	1.89	*Apoa4*	−0.95	−0.95
*Isg15*	1.21	-	*Ribc2*	−0.96	−0.9
*LOC690082*	1.19	1.03	*Selenop*	−0.97	−0.96
*Aqp3*	1.17	1.36	*Aoah*	−1.01	-
*Capn3*	1.12	1.16	*Cyp3a62*	−1.03	−1.2
*Rasa4*	1.11	1.07	*Sla2*	−1.03	-
*Tmem52*	1.1	-	*Fcgrt*	−1.08	−1.21
*Fyb2*	1.07	1.05	*LOC102555026*	−1.09	−1.67
*Chdh*	1.07	1.24	*Igfals*	−1.11	−1.35
*Samd9*	1.06	1	*Gpx3*	−1.13	−1.32
*Upk1b*	1.02	1.21	*Ephb6*	−1.13	−1.2
*Samd9*	0.99	-	*Cd36*	−1.17	−1.1
*Ces1e*	0.93	0.92	*LOC691352*	−1.2	−1.11
*MGC108823*	0.91	0.92	*Ankrd29*	−1.23	−1.44
*Oas1i*	0.9	-	*Ptprr*	−1.23	−1.28
*Mcpt4*	0.9	-	*Hoxc11*	−1.26	−1.41
*LOC100912658*	0.86	-	*Tacr3*	−1.31	−1.63
*Lgals3bp*	0.83	-	*Srd5a2*	−1.32	-
*Slc37a4*	0.73	0.83	*C8g*	−1.52	−1.53
			*Dapl1*	−1.54	−1.82
			*Ggh*	−1.58	−1.49
			*Slc3a1*	−1.63	−1.09
			*Pdx1*	−1.66	−1.8
			*Kcne1*	−2.33	−2.63
			*Aqp8*	−2.35	−2.85
			*RGD1562699*	−2.66	-
			*Afp*	−3.29 *	−5.39
			*Onecut2*	−3.75	−3.71
			*Tmprss15*	−5.13	−5.86

Results are expressed as fold change. All genes in the table are significantly modified in the RV group vs. the REF group. (-) non-modified compared to REF. * *p* < 0.05 compared to REF (*n* = 4/group).

## Data Availability

Data sets generated can be obtained upon request from the authors.
